# Radiological evidence in legal contexts: forensic versus clinical reporting in cases involving living victims of violent crime

**DOI:** 10.1007/s12024-026-01215-8

**Published:** 2026-03-18

**Authors:** Ingrid I.H. Haest, Bernice Oude Grotebevelsborg, Hans Nelen, Harry van Venrooij, Marijke Buiskool, Marko Jelicic

**Affiliations:** 1 Department of Criminal Law and Criminology, Faculty of Law, Maastricht University, Maastricht, P.O. Box 616, 6200 MD the Netherlands; 2 Netherlands Forensic Institute, The Hague, P.O. Box 24044, 2490 AA Netherlands; 3 Public Health Service, Department of Forensic Medicine, Amsterdam, P.O. Box 2200, 1000 CE the Netherlands; 4 Forensic Psychology Section, Department of Clinical Psychological Science, Faculty of Psychology and Neuroscience, Maastricht University, P.O. Box 616, Maastricht, 6200 MD the Netherlands

**Keywords:** Forensic science, Magnetic resonance imaging, MRI, Computed tomography, CT, Clinical radiology report, Forensic radiology reports, Clinical forensic imaging

## Abstract

**Purpose:**

Forensic medical examinations are essential for evaluating victims of potential crimes. Radiology, particularly computed tomography (CT), has become a valuable tool in postmortem and clinical forensic contexts. However, when assessing living victims, forensic evaluations often face challenges due to reliance on clinical records, which primarily focus on medical treatment rather than forensic interpretation. Clinical radiology (CR) reports frequently omit forensically relevant findings. In contrast, forensic radiological (FR) reports may provide more relevant insights into such details, although, this distinction has not been systematically studied. The aim of the study was to evaluate the forensic relevance of CR reports compared to FR reports, with the goal of exploring and understanding potential benefits of involving forensic radiologists in legal investigations.

**Methods:**

This retrospective study compares CR and FR reports concerning living victims of serious violence in the Netherlands between 2016 and 2022. Cases were included if both report types were available. Three experts reviewed the cases, and a paired samples test was used to assess differences.

**Results:**

A total of 45 forensic cases were analyzed, including blunt force injuries, stabbings and gunshot wounds. FR reports consistently outperformed CR reports in answering case-relevant forensic questions. In 16 cases with identical FR and CR scores, notable discrepancies were observed, particularly regarding soft tissue findings and skin lesions, which were more comprehensively described in FR reports.

**Conclusion:**

This study shows that the forensic re-evaluation of clinical imaging in potential crime victims yields more forensically relevant information than the CR reporting.

## Key points

1. Clinical radiology reports frequently omit forensically relevant findings.

2. Forensic radiology reports provide significant more answers for the forensic relevant question.

3. In forensic radiology reports soft tissue abnormalities are described in greater detail than in the clinical radiology reports.

4. Forensic re-evaluation of clinical imaging in potential crime victims yields more forensically relevant information than the clinical radiology reports.

## Introduction

Forensic science is a multifaceted discipline that integrates various scientific methods and technologies to contribute to aid in criminal investigations and legal proceedings. Whenever a potential criminal act results in injury to a victim—either living or deceased—a comprehensive forensic medical examination is essential to support the forensic investigation conducted by the public prosecutor and the police [1]. The public prosecutor requires such information to determine the course of the investigation and to inform decisions regarding prosecution, while the police use it to guide forensic technical investigations. Depending on the nature of the injuries, various questions may be posed to the forensic medical examiner by the public prosecutor or the police.

One emerging area within forensic medical science is radiology, which involves the use of medical imaging techniques to examine the human body. Traditionally associated with diagnosing and treating medical conditions, these imaging techniques have significantly evolved to also play a significant role in forensic investigations. Today, radiology is considered an essential diagnostic tool with a wide range of applications within postmortem investigations [2, 3]. However, radiological techniques—particularly computed tomography (CT)—are equally valuable in assessing living individuals who have sustained injuries resulting from a possible criminal act [4]. Due to advances in medical treatment, victims of crimes may survive if they are treated promptly in a hospital. In a clinical setting, severe injuries are documented in medical records, and imaging procedures such as CT or digital X-rays are frequently performed.

During postmortem examinations, several forensic medical procedures may be performed, including external examination, toxicology, radiology, and autopsy. The external forensic assessment of inflicted injuries in living victims involves physical examination, visual inspection, and documentation and photography of external injuries—often crucial to a forensic investigation. Describing skin lesions, bruises, or other injuries can be important to determine the circumstances surrounding the acts of violence. However, external forensic examination offers limited insights, as it cannot fully provide insight into accompanying internal injuries [5]. Another limitation is that, in many cases involving serious injuries, a timely forensic medical examination is not possible. The forensic medical examination may be delayed by several days, weeks, or even months after the incident. This delay may occur when the victim cannot be examined for medical reasons—such as emergency surgery—or when the patient is unable to provide informed consent, and no legal representative is available. By the time a forensic medical examination is conducted, it may be difficult to accurately assess the initial injuries. Wound healing, medical treatment, and/or complications from the primary injury may complicate forensic medical interpretation. In such cases, the interpretation of the injuries often relies on documentation, such as medical records.

A significant limiting factor for a thorough forensic medical examination is that medical records usually contain only clinically relevant findings and often lack forensically relevant findings. Findings of minor clinical relevance, such as superficial cuts, bruises, or swelling, are frequently omitted [6]. Additionally, most clinical physicians lack expertise or training in forensic injury interpretation. This limitation extends to clinical radiologists as well. Clinical radiology (CR) reports, often included in medical records, are written to gain insight into a patient’s condition for medical treatment. Because clinical radiologists are generally unfamiliar with forensic protocols, the use of their radiological reports is of limited value in the context of forensic investigations. Therefore, radiological reports should ideally be written by forensic radiologists who have expertise in writing court reports [7].

CR reports typically do not address specific forensic questions, such as distinguishing between injuries caused by blows versus falls or identifying the type of weapon used [7]. Therefore, re-evaluating CT images by a forensic radiologist can be highly beneficial in establishing a medico-legal assessment.

In forensic cases, routine imaging performed upon hospital admission may serve as the sole source of morphological evidence. In case images are not immediately used in a forensic investigation, they can still be stored and accessed later, allowing multiple image reviews. Furthermore, such images can be reconstructed into three-dimensional (3D) images and used to explain complex cases to non-medical professionals involved in the forensic investigations. In the Netherlands, a conviction for aggravated assault is only possible in cases where it has been established that a victim sustained serious physical injuries caused by the defendant’s actions [8, 9]. To prove aggravated assault, the judge must be able to attain a comprehensive understanding of the physical injuries. Expert testimony regarding injuries in living victims based on a CR report may underestimate the severity of the injuries and typically lacks 3D CT reconstructions [4]. For that reason, this type of testimony is of limited value to a judge [6]. There has been no systematic research into the value of a forensic radiological (FR) report compared to a CR report in cases involving living victims of possible criminal acts.

Therefore, it is important to investigate whether a FR report derived from clinical data provides sufficient information to address forensic questions. In the Netherlands, forensic evaluations of clinical data are not routinely requested.

The present study examines the extent to which forensically relevant information is included in CR reports drafted at hospital admission for victims of serious violent crimes, compared with information found in FR reports drafted by radiologists trained in forensic reporting. The goal of this comparison is to assess the possible added value of the latter.

## Materials and methods

### Material

This is a retrospective study involving living victims who sustained serious injuries potentially resulting from a criminal act. The victims were examined at various hospitals across the Netherlands. The study material consists of CR and FR reports collected over a six-year period, from 2016 to 2022. All cases were selected from the case records of the Netherlands Forensic Institute (NFI). All Dutch cases involving both an FR report and a CR report about living victims aged 18 years or older were included. Both the CR and FR report authors had access to the full clinical imaging dataset, and both reporters could use multiplanar reconstructions (MPRs) at their discretion. The use of CR and FR reports in this study was reviewed and approved by the Maastricht University Institutional Review Board, which determined that the research qualified for exemption under Dutch Medical Research Involving Human Subjects Act (WMO). All patient data were fully anonymized prior to analysis to protect confidentiality; any identifying information was removed or coded in accordance with institutional and legal guidelines. Due to the retrospective and anonymized nature of the data, the requirement for informed consent was waived by the Maastricht University Institutional Review Board.

### Methods

This study employs a retrospective data analysis. Cases were evaluated based on questions formulated by the forensic physician during the initial request for re-assessment. The questions were incident type specific, for a specific incident type, for each case the same questions were asked. General questions typically included, what (traumatic) abnormalities can be observed based on the radiological data? Where are they located, and, if possible, provide a 3D image? The questions regarding blunt force trauma were: can a (substantiated) statement be made regarding the (minimum) number of instances blunt force was applied, the location of the application of these force(s), and if there was any indication of coup-contrecoup injury? This last question was only asked in cases with possible head trauma. The questions in stab and gunshot wound cases concerned the location of the skin lesions, the dimensions of any skin perforations or lesions, the direction of the wound trajectories, the proximity to vital organs and therefore potential life-threatening situations and, the location of any metal-containing foreign bodies. The FR report authors had no additional clinical or forensic data. The number of questions per case ranged from 1 to 12, with a mean of 4.4.

The answers were assessed and scored according to the following criteria:

- Two points (2) for an answer.
- One point (1) for an incomplete answer (the answer did not cover all aspects).
- Zero points (0) for an absence of an answer.
- Minus one point (− 1) if the body part referred to in the question was not imaged.

To achieve consensus among the content experts on how to rate the answers on forensically relevant questions based on CR and FR reports, six reports were independently rated and subsequently discussed by the experts.

Three content experts—two medical forensic examiners (MD’s) and one forensic examiner (LL.M), each with more than 10 years of relevant experience in complex medical forensic casework, including at NFI—independently evaluated each CR and FR report in a given case. Any discrepancies in scoring were resolved during a set of consensus meetings. Questions rated − 1 were excluded from the evaluation, as they did not contribute to answering the research question. The maximum score achievable per report is twice the number of questions. The final score for a report was calculated as ten times the total number of points obtained, divided by the maximum possible points. If this score was less than 0, it was replaced by 0. In this manner, a score was assigned within the interval between 0 and 10, adjusted for differences in the number of questions per case. The content experts were blinded for the origine of the reports, however based on the content of each report it was immediately obvious whether it was a CR or FR report. The content experts had no access to the image date, only the reports were rated. Interrater reliability was assessed using the intraclass correlation coefficient (ICC). A two-way mixed-effects model with absolute agreement was used, treating raters as fixed effects and subjects as random effects. Reliability was calculated for both single ratings and the mean of the three raters. To assess the magnitude of the observed differences between the scores of the various report types, a paired samples test and a one-way analysis of variance were applied. The choice between parametric and nonparametric statistical tests was based on the assessment of data normality using the Shapiro-Wilk test. Effect sizes were calculated using Cohen’s d. All analyses were conducted with IBM SPSS Statistics, version 30.0 (IBM Corp., Armonk, NY, USA).

In cases with an identical score, the discrepancies in the forensic-relevant information in both reports were assessed.

## Results

A total of 45 cases met the predefined inclusion criteria. The cohort comprised 30 male and 15 female victims, with a mean age of 35 years (range: 18–83 years). The mean interval between the CR and FR was 8 months (range: 1 to 74 months; SD = 12.9). The predominant types of traumas were blunt force injuries (*n* = 22), stab wounds (*n* = 15), and gunshot wounds (*n* = 7). One case involved a victim with thermal injury. Imaging modalities included CT in 40 cases, conventional radiography (plain X-rays) in 36 cases, and magnetic resonance imaging in 10 cases. The distribution is detailed in Table 1.

**Table 1:** Distribution of the applied medical imaging techniques

# cases	X-ray	CT	MRI
26	✓	✓	X
6	✓	✓	✓
6	X	✓	X
3	✓	X	X
2	X	✓	✓
1	✓	X	✓
1	X	X	✓

A score of minus one was recorded for only one question in a single case, consequently, this data point was excluded from further analysis.

Interrater reliability of the scoring of the CR reports among the three raters was good for single ratings, ICC (3.1) = 0.77, 95% CI [0.72, 0.81], *p* < .001, and excellent for the mean of the three raters, ICC (3.3) = 0.91, 95% CI [0.88, 0.93], *p* < .001. For the scoring FR reports the interrater reliability was moderate for single ratings, ICC (3,1) = 0.56, 95% CI [0.48, 0.63], *p* < .001, and good for the averaged ratings, ICC (3.3) = 0.79, 95% CI [0.73, 0.84], *p* < .001. A Shapiro–Wilk test indicated a significant deviation from normality for both the scores of the CR and FR (W=0.898, *p*<.001 and W=0.911, *p* = .002). Therefore, a linear regression model was used for further analysis.

A linear regression model predicting the paired difference score (FR score - CR score) was statistically significant, F (2.42) = 47.17, *p* < .001, explaining 69% of the variance (R² = 0.69). At the average number of questions per case, the adjusted mean paired difference was 4.04 points (95% CI [1.98, 6.10], *p* < .001), indicating higher FR scores than CR scores. The number of questions per case was positively associated with the paired difference (B = 1.55, *p* < .001), whereas incident type was not a significant predictor. Imaging modality was excluded from the final model because it was not significantly associated with the paired difference and did not materially change the estimated effect. The effect size, measured by Cohen’s d, was large (d = 0.86).

For the cases in which the FR and CR reports received identical scores, discrepancies in the forensically relevant information were assessed to determine whether the reports could be considered interchangeable.

In 16 cases, both the CR and FR reports received the same score. Of which, 6 cases with no incident related abnormalities, showed no essential discrepancies between the two reports. Seven of the remaining cases were blunt force (BF) incidents where the questions were limited to descriptions of traumatic abnormalities and the location thereof. While these abnormalities were described in both reports, no assessment was made as to whether all abnormalities were described; however, it was noted that FR reports generally included a more extensive description of abnormalities. In these BF cases, the FR reports described more soft tissue abnormalities and in greater detail. In six of these cases, soft tissue findings were not mentioned in the CR reports, and in one CR report, the soft tissue abnormalities were described with less detail. Additionally, in two of the seven BF cases, CR reports omitted any mention of skin lesions, and in one case, old and small fractures—though clinically insignificant—were not described in the CR report.

Three cases that received identical scores were perforating injuries (PP) incidents related. In these cases, the questions addressed the dimensions of skin lesions, wound trajectories, and the presence of metallic foreign bodies. In one case, more wound trajectories were described in the FR report than in the CR reports. In a second case, the injuries caused by the penetrating trauma were described in both reports. However, in the FR report more abnormalities were identified than in the CR report, including soft tissue swelling and a wound trajectory, even though this was not specifically requested. In a third case, both reports provided the same information, but neither included the dimensions of wounds. Only one trajectory was described in both reports. A notable observation was that in this third case, the CR report mentioned the entry and exit wounds, whereas in the FR report this differentiation was not made, noting that the trajectory passed through soft tissues only.

## Discussion

The main finding of this study is that FR reports addressed significantly more forensically relevant questions than CR reports across all trauma types. FR reports showed no significant difference in the number of questions answered between PP and BF cases. Differences between CR and FR scores are influenced by case complexity; in more complicated cases with a higher number of forensic-relevant questions, the discrepancy between CR and FR reports was larger.

Interrater reliability was good to excellent for the scoring of the CR reports, likely reflecting their limited complexity with regard to forensically relevant information. In contrast, FR reports showed moderate agreement for single ratings and good agreement when ratings were averaged, consistent with the greater interpretative complexity of FR reports.

To support a medical forensic examination, it is essential to gather all possible evidence related to injuries sustained by victims of serious crimes. For victims receiving treatment in a hospital, medical imaging techniques are often used to assess the severity of injuries. The choice of imaging modality is often driven by the clinical question and the associated time investment [10]. As a result, the selected modality may not align with the preferences or requirements posed by forensic investigative questions. Additionally, imaging quality and technical parameters related to collimation are of importance in forensic settings, especially for the visualization of soft tissues. Although soft tissue findings have evidentiary value, subcutaneous soft tissues are often excluded from imaging due to radiation protection measures and image quality considerations. Despite these limitations, an FR report on a victim of a serious crime remains valuable to get answers on what happened.

In cases where no incident related abnormalities were described in either the FR or CR report, the number of answered questions was identical, and these reports were considered interchangeable. In all other cases where the scores were the same, the FR report provided more relevant forensic information. This study identified considerable underreporting of lesions in CR reports. This observation is consistent with the findings of Schuh et al., who reported that clinical radiologists identified fewer abnormalities on clinical CT compared to their forensic counterparts [11]. The present study also suggests that such omissions are, in part, due to the clinical radiologist’s lack of familiarity with forensic priorities and, more importantly, the fact that their reporting is primarily aimed at providing clinically relevant information for the acute management of the victims. Because of the fundamentally different interpretations of identical imaging material by clinical and forensic radiologists, discrepancies arise in their reports, with clinical radiologists also tending to underreport findings in forensic cases [6]. Forensic radiologists receive specific training to include clinically insignificant injuries and provide more detailed descriptions in their assessments, as such findings may hold relevance within a medico-legal framework.

Previous studies have shown that, in acute settings, CT is frequently utilized due to its practicality, specificity, and sensitivity in detecting traumatic bone injuries, as well as injuries to solid organs, hollow viscera, lungs, and vascular structures [10]. Similarly, forensic literature supports the frequent application of CT in postmortem investigations and highlights its ability to address a wide range of forensic questions. However, as demonstrated in the present study, as well as other studies, it is always beneficial to have forensic radiologists perform a secondary evaluation of the images, regardless of the modality used [11, 12].

Even though CT is both specific and sensitive in detecting traumatic abnormalities, not all forensically relevant information is included in the CR report. This is illustrated by the following excerpts from the CR and FR report of a case involving a stabbing incident.

CR report: “Stab wound on the right dorsal side at the level of the 12th rib. There is a significant hematoma in the right retroperitoneal space. Within the hematoma, a small air bubble and a small blush are visible, most likely originating from the intercostal artery of the 12th rib. Additionally, there is a small subcapsular hematoma dorsally on the right kidney, consistent with a grade 1 renal injury. There is no evidence of urinary extravasation or hydronephrosis.”

FR report: “From the skin defect on the right side of the back, a trajectory can be followed reaching the lower pole of the right kidney. The trajectory runs from back to front, from left to right, and slightly upwards. Radiologically, this trajectory has a minimum length of approximately 10 cm. The retroperitoneal hematoma has a thickness of approximately 2 cm; the minimal length of the trajectory is therefore approximately 8 cm. The contrast enhancement in the soft tissues beneath the skin defect is, with near certainty, caused by active bleeding from a lumbar artery. The fluid (blood) in the right perirenal space is, with near certainty, caused by the penetrating trauma.”

**Fig. 1:**
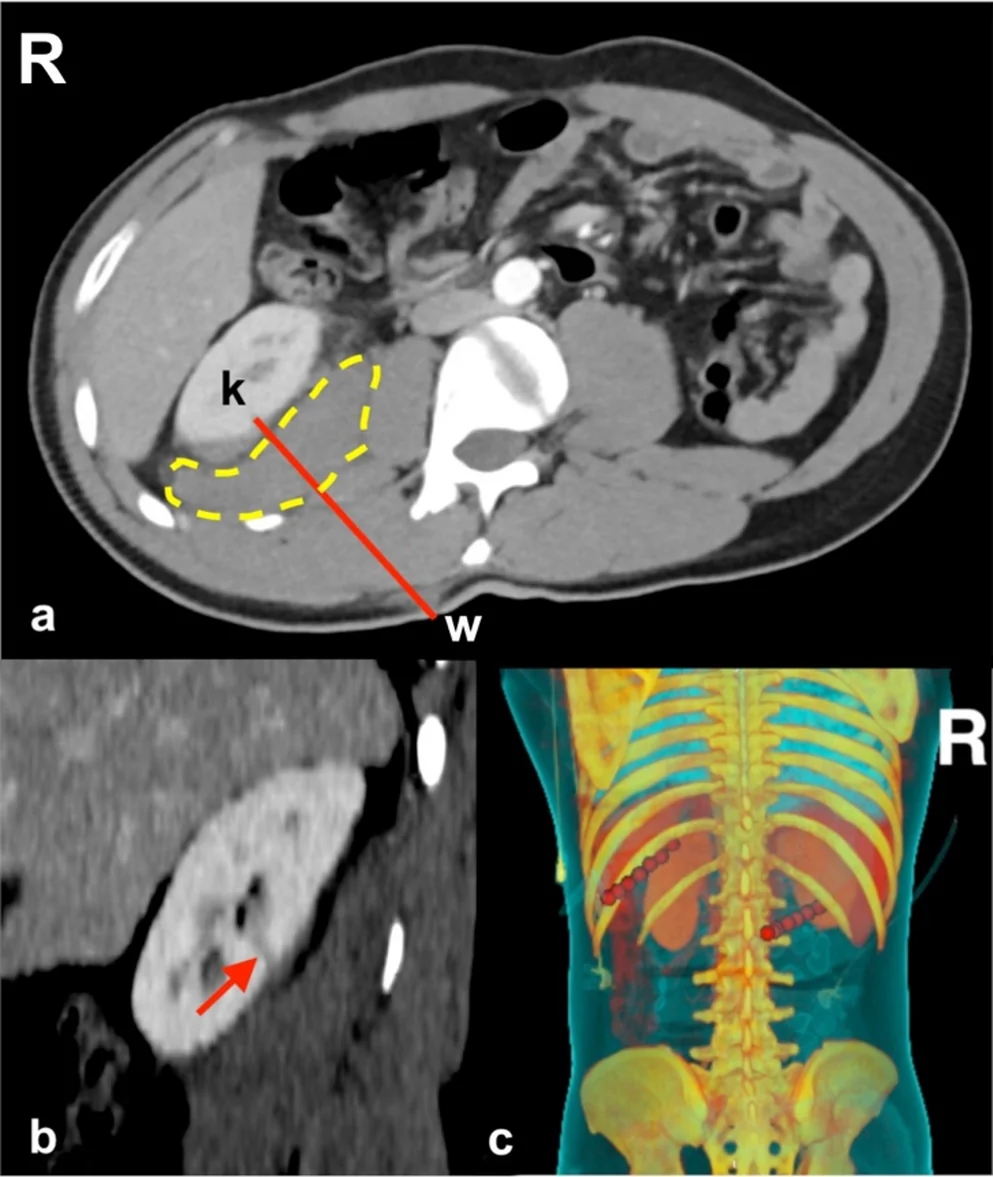
Clinical computed tomography (CT) imaging of a patient following stab wounds. Axial CT image demonstrating a cutaneous defect, with the trajectory (red line) of one stab wound extending into the right kidney. Associated retroperitoneal hemorrhage is also shown (yellow line). (**a**) Sagittal reconstruction shows the course of the penetrating injury in the right kidney (arrow). (**b**) Three-dimensional posterior view of the torso illustrates the direction and spatial orientation of two wound trajectories in this victim with red markers. (**c**) k = kidney, w = wound, R = right side

The FR report was supplemented with 2D and 3D images to illustrate the findings (Fig. 1).

From a clinical perspective, parameters such as wound size or trajectory length may be of limited interest. However, from a forensic viewpoint, such details may provide crucial information regarding the characteristics or dimensions of a weapon used [13].

Additionally, not all reports clearly stated whether metallic foreign particles remained present in the body. Other medical specialists, such as surgeons or emergency physicians, do not routinely request 3D imaging in clinical practice. However, this was specifically requested by the forensic examiner, as literature suggests that three-dimensional reconstructions facilitate improved understanding by the judiciary [14, 15]. These visualizations may assist in determining the shape and dimensions of the weapons used, thereby contributing significantly to the evidentiary process.

Because all cases were selected from the files of the Netherlands Forensic Institute there might have been a selection bias toward serious or complicated cases, both with respect to medical and legal issues. However, also relatively simple single blunt-force cases were included. Although all effort was made to blind the content experts for the origine of the reports, based on the content of each report it was immediately obvious whether it was a CR or FR report. This could introduce a bias, however, the content experts assessed quality of the answers on the factual data available in both reports.

A key strength of this study is that it analyzed a series of cases involving various types of incidents. To the authors’ knowledge, this is the first study to provide such a comprehensive comparative evaluation.

Finally, as clinical imaging primarily serves hospital-based physicians, standard clinical radiological reports are typically written with therapeutic intent. In contrast, forensic radiological reports used in legal proceedings must be accurate, concise, and comprehensible to the judiciary, as emphasized in the existing literature [10]. Moreover, previous studies indicate that three-dimensional reconstructions can enhance judicial understanding of radiological findings [14, 15]. These differences highlight the importance of re-evaluating imaging findings by a forensic radiologist to ensure that reports meet forensic standards.

Given the potential selection bias and limited sample size of the current study, broader implementation may help generate additional cases for further research evaluating forensic impact and cost-effectiveness before routine or mandatory adoption is recommended.

## Conclusion

This study demonstrates that forensic re-assessment of clinical imaging in victims of suspected criminal acts can yield additional forensically relevant information compared with standard clinical radiology reports. The findings support considering forensic radiological re-assessment.

## References

[1] Wiersema J, Pierce M, Woody A, Sanford M. Evidence Management Best Practices in Medicolegal Death Investigation. In: Mozayani A, Fisher CP, editors. Forensic Evidence Management. New York: Taylor & Francis Group; 2018. pp. 55–75.

[2] Brogdon BG. Introduction to forensic radiology. In: Thali M, Viner M, Brogdon BG, editors. Brogdon’s Forensic Radiology. Boca Raton Taylor & Francis Group; 2011. pp. 3–23.

[3] Flach PM, Thali MJ, Germerott T. Times Have Changed! Forensic Radiology—A New Challenge for Radiology and Forensic Pathology. AJR Am J Roentgenol. 2014;202(4):325–34. 10.2214/AJR.12.10283 24660730

[4] Borowska-Solonynko A, Solonynko B. The use of 3D computed tomography reconstruction in medico-legal testimony regarding injuries in living victims - Risks and benefits. J Forensic Leg Med. 2015;30:9–13. 10.1016/j.jflm.2014.12.006 25623187

[5] Hassler EM, Ogris K, Petrovic A, Neumayer B, Widek T, Yen K, et al. Contrast of artificial subcutaneous hematomas in MRI over time. Int J Legal Med. 2015;129(2):317–24. 10.1007/s00414-014-1124-8 25416961

[6] Heimer J, Arneberg L, Blunier S, Klukowska-Rötzler J, Gonzenbach AG, Exadaktylos A, Ruder T, Wagner F. Under-reporting of forensic findings: craniocervical emergency imaging in cases of survived hanging. Forensic Science, Medicine, and Pathology. 2024;20(2):434–42. 10.1007/s12024-023-00665-8 PMC11297113 37340278

[7] Bauer M, Polzin S, Patzelt D. The use of clinical CCT images in the forensic examination of closed head injuries. J Clin Forensic Med. 2004;11(2):65–70. 10.1016/j.jcfm.2003.10.003 15261000

[8] Supreme Court, 3th July 2018, ECLI:NL:PHR:2018:718.

[9] District Court Middle-Netherlands. 2nd Februari 2023, ECLI:NL:RBMNE:2023:3844.

[10] Dedouit F, Ducloyer M, Elifritz J, Adolphi NL, Yi-Li GW, Decker S, et al. The current state of forensic imaging– clinical forensic imaging. Int J Legal Med. 2025;139(4):1639–46. 10.1007/s00414-025-03464-8 PMC12170677 40100352

[11] Ruder TD, Gonzenbach A, Heimer J, Arneberg L, Klukowska-Rötzler J, Blunier S, et al. Imaging of alert patients after non-self-inflicted strangulation: MRI is superior to CT. Eur Radiol. 2024;34(6):3813–22. 10.1007/s00330-023-10354-3 PMC11166758 37953368

[12] Schuh P, Scheurer E, Fritz K, Pavlic M, Hassler E, Rienmüller R, et al. Can clinical CT data improve forensic reconstruction? Int J Legal Med. 2013;127(3):631–8. 10.1007/s00414-013-0830-y 23412132

[13] De Bakker BS, Soerdjbalie-Maikoe V, De Bakker HM. The use of 3D-CT in weapon caused impression fractures of the skull, from a forensic radiological point of view. J Forensic Radiol Imaging. 2013;1(4):176–9.

[14] Woźniak K, Rzepecka-Woźniak E, Moskała A, Pohl J, Latacz K, Dybała B. Weapon identification using antemortem computed tomography with virtual 3D and rapid prototype modeling-A report in a case of blunt force head injury. Forensic Sci Int. 2012;222:1–3. 10.1016/j.forsciint.2012.06.012 22748480

[15] Wittschieber D, Beck L, Vieth V, Hahnemann ML. The role of 3DCT for the evaluation of chop injuries in clinical forensic medicine. Forensic Sci Int. 2016;266:e59–63. 10.1016/j.forsciint.2016.05.025 27287510

